# A Randomized, Blinded, Placebo‐Controlled Crossover Study of the Pharmacokinetics and Pharmacodynamics of Naloxone, Naltrexone, and Nalmefene in Methadone‐Sedated Working Dogs

**DOI:** 10.1111/jvp.13515

**Published:** 2025-05-08

**Authors:** Travis Mills, Mary A. Robinson, Ciara Barr, Darko Stefanovski, Youwen You, Rachel Proctor, Kasey Seizova, Amritha Mallikarjun, Cynthia M. Otto

**Affiliations:** ^1^ Department of Clinical Sciences and Advanced Medicine, School of Veterinary Medicine University of Pennsylvania Philadelphia Pennsylvania USA; ^2^ Department of Clinical Studies ‐ New Bolton Center, School of Veterinary Medicine University of Pennsylvania Kennett Square Pennsylvania USA; ^3^ PA Equine Toxicology and Research Laboratory West Chester Pennsylvania USA; ^4^ Penn Vet Working Dog Center, School of Veterinary Medicine University of Pennsylvania Philadelphia Pennsylvania USA

**Keywords:** drug detection dog, mu antagonist, narcotization, opioid, reversal

## Abstract

A randomized, blinded, placebo‐controlled crossover study was performed with eight professional working dogs to evaluate the pharmacokinetics and pharmacodynamics of three opioid reversal agents. Following sedation with 1 mg kg^−1^ methadone HCl IV, dogs were randomly assigned to receive naloxone, naltrexone, or nalmefene at 0.1 mg kg^−1^ IM, or saline (0.1 mL kg^−1^). Sedation scores and vital signs were obtained for 6 h, and blood samples were obtained for 72 h. Across all groups and phases, the mean sedation score prior to methadone was 0.93/14, and prior to reversal/placebo was 11.45/14. Mean sedation scores 1 min post reversal/placebo were 7.6, 7.4, 8.1, and 10.6; and at 5 min were 2.2, 1.9, 1.9, and 11.8 for nalmefene, naloxone, naltrexone, and saline, respectively. Dogs with a sedation score ≥ 10/14 at 20 min after reversal/placebo were administered 0.1 mg kg^−1^ of naloxone IM and included all dogs that received saline. The reversal agents significantly decreased sedation scores within 5 min (*p* < 0.001) and reversal was maintained for the duration of the study. A previously undetected slower terminal elimination phase was observed for all analytes between 24 and 72 h; however, future studies with additional time points between 6 and 24 h are needed to generate pharmacokinetic estimates for this phase. These reversal agents may be useful for treating sedation in professional working dogs exposed to opioids prior to seeking veterinary care. Pharmacological differences between methadone and higher potency opioids (fentanyl and its derivatives) preclude interpretation of these findings to non‐methadone opioids, and further studies are needed.

**Abbreviations**
*f*_r_
: respiratory rate
HR: heart rate
IM: intramuscular
IV: intravenous
MAP: mean arterial pressure
SpO^2^
: pulse oximetry

## 1 Introduction

Working dogs, especially drug‐detection dogs, are at an increased risk of exposure to potent or ultrapotent opioids such as fentanyl or carfentanil due to increased illicit manufacturing of these opioids and drug trafficking (Palmer and Gautier 2017). In the clinical setting, reversal of opioid‐induced sedation and behavioral changes can be achieved by the administration of a μ‐opiate receptor antagonist, such as the μ‐antagonists naloxone or naltrexone, or the κ‐agonist μ‐antagonist butorphanol (Pascoe 2000). In the field setting, naloxone in the form of NARCAN is the μ‐antagonist typically available to working dog handlers; however, a new injectable product containing the μ‐antagonist nalmefene has recently been approved for clinical use in humans.

Naloxone has been demonstrated to be an effective reversal agent when administered intramuscularly or intranasally for reversing fentanyl‐induced sedation in dogs (Barr et al. 2023; Freise et al. 2012). However, its efficacy for reversing longer‐acting opioid agents, such as methadone, is unknown. Previous studies have suggested that nalmefene is approximately four times more potent than naloxone and may be better for preventing re‐narcotization in dogs (Wilhelm et al. 1995). Re‐narcotization is the return of clinical signs after the μ‐antagonist falls below clinically effective concentrations while the inciting agent is still of sufficient concentration to have negative effects. Effects of methadone administration in dogs may include sedation, panting, respiratory depression, vomiting, defecation, or dysphoria (characterized by whining/whimpering, agitation, or thrashing behavior; Becker et al. 2013). These effects in working dogs in a field setting are undesirable and may compromise their welfare.

Given the increased exposure risk working dogs face, the goal of this study was to investigate if there were differences in the pharmacokinetics or pharmacodynamics of three commercially available reversal agents administered by the intramuscular (IM) route when dogs were administered the longer‐acting opioid, methadone. Based on previous studies, naltrexone and nalmefene were hypothesized to be more efficacious than naloxone. A longer duration of antagonism by either naltrexone or nalmefene than naloxone would be beneficial in a field exposure scenario to allow a handler to reach veterinary attention following working dog exposure.

## 2 Materials and Methods

### 2.1 Animals

This protocol was approved by the Institutional Animal Care and Use Committee at the University of Pennsylvania (Protocol # 807164) and the Army Animal Care and Use Review Office Proposal (Number 77711‐ST). This was a randomized, blinded, crossover design with at least a 2‐week wash‐out period in between phases. Eight working dogs from the Penn Working Dog Center comprised of German Shepherds (*n* = 2), Labrador Retrievers (*n* = 4), a Dutch Shepherd (*n* = 1), and a Small Munsterlander (*n* = 1) were selected for the study. All dogs were University‐owned and participated in detection training or specific research projects 5 days a week but lived with foster families on evenings and weekends. Dogs enter the program at 8 weeks of age and graduate from the program at approximately 12–18 months to become professional working dogs (e.g., law enforcement, search and rescue, or single purpose detection), or join the long‐term research program, or are released from the program as pets. A priori inclusion criteria were age > 8 months, comfort with being handled, and completing the low stress husbandry training. Dogs that were < 8 months, fearful or unwilling to stand for blood collection, or had any systemic disease based on physical examination were excluded. Four dogs were female and four were male. At the start of the study, the ages ranged from 8.8 to 68.7 months (median 12.6 months). The dogs were weighed prior to each trial. All dogs were assessed to be healthy based on physical examination by a veterinarian prior to each trial. Dogs in the Penn Vet Working Dog Center program undergo routine husbandry training as part of their detection dog training; however, this group of dogs underwent additional training during their normal workdays at the Center. Prior to the study, all dogs received at least 10 sessions of husbandry training including desensitization to tactile stimulation at the catheter site, desensitization to noise made by hospital equipment, desensitization to blood pressure cuffs and stethoscopes, and restraint training.

### 2.2 Study Design

**FIGURE 1:**
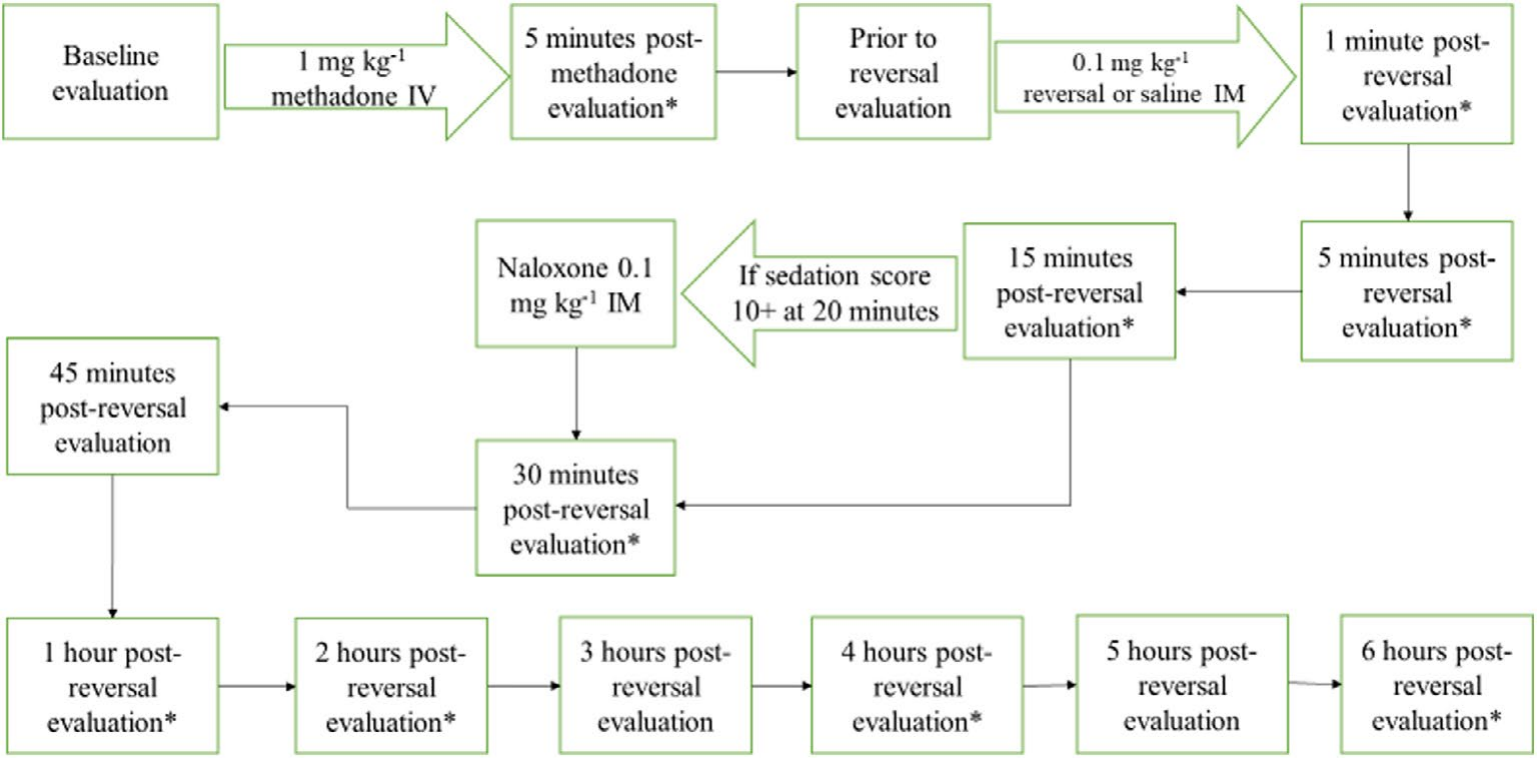
Schematic indicating timing of evaluations and blood collection from the initiation of the trial through 6 h. Evaluation included heart rate, respiratory rate, blood pressure measurement, and sedation score acquisition. * indicates blood collection.

All dogs were fasted from 20:00 the evening prior to the study but had free access to water. Foster families dropped off their dog by 07:30 and picked up their dog at the end of the day. The study began at 08:00. The eight dogs were divided into two groups of four each to allow four dogs to be tested per day. A phase consisted of testing all eight dogs over two consecutive days. Each dog underwent four trials, one in each phase, except for one dog which was removed from the study following phase II. For each trial, dogs were sedated with methadone (1.0 mg kg^−1^; Methadone hydrochloride, 10 mg mL^−1^; Akorn, Lake Forest, Illinois) intravenously (IV) via a 19 gauge butterfly catheter (BD, Franklin Lakes, New Jersey) in either a cephalic or saphenous vein. Once sedated, an 18 or 20 gauge over‐the‐needle catheter (BD Insyte, Franklin Lakes, New Jersey) was placed in a cephalic or saphenous vein to allow blood sampling for pharmacokinetic analysis of drug plasma levels. Heart rates, oscillometric blood pressure, pulse oximetry, and respiratory rates (*f*_*r*_) were monitored immediately prior to sedation, 5 min after sedation, immediately prior to reversal, 1, 5, and 15 min post‐reversal, and then every hour for 6 h post‐reversal (see Figure 1). On each trial day, the dogs were housed in kennels when they were not being evaluated. Each dog was separately walked on a leash into the building for evaluation during each evaluation period. Once the evaluation and blood collection (if necessary) were complete, they were walked back to a nearby individual cage until the next evaluation period. Heart rate was confirmed via auscultation and pulse palpation, and respiratory rate was obtained by observing the chest wall excursion. The Masimo Rad‐57 pulse CO‐oximeter (Masimo; Irvine, California) was used to measure SpO_2_, and the PetMap+II+ Multiparameter (Ramsey Medical; Tampa, Florida) was used to measure blood pressure and SpO_2_. Equipment maintenance was performed according to manufacturer directions. Adverse events including bradycardia (heart rate < 60 beats per minute), hypotension (mean arterial pressure < 60 mmHg), respiratory depression (pulse oximetry values < 95%) and vomiting or regurgitation were monitored and recorded. Dogs had supplemental oxygen supplied via facemask at 5 L min^−1^ if they were deemed to be hypopneic (*f*_r_ < 16), with pulse oximetry readings of an SpO^2^ < 95%, or if a pulse oximetry reading could not be obtained while the dog was sedated.

For each of four phases, dogs were randomly assigned to receive 0.1 mg kg^−1^ of either naloxone hydrochloride (Naloxone hydrochloride 1 mg mL^−1^; IMS limited, So El Mote, CA), naltrexone hydrochloride (Trexonil Wildlife Pharmaceuticals; 1230 West Ash, Windsor, CO 80550), nalmefene base (Nalmefene hydrochloride 1.108 mg/mL, Purdue Pharma L.P., Stamford, CT), or an equal volume of saline (0.1 mL kg^−1^) in the left lumbar epaxial muscle (IM) 10 min following methadone administration. Only the one investigator who drew up the doses (CMO) was unblinded to the agent; all other participants were blinded. The washout period between phases was at least 2 weeks.

During each phase, four dogs were tested 1 day (Group A) and the remaining four dogs were tested the following day (Group B). Dogs were initially randomized into group A or B using a random number generator. A full block randomization across two groups for age and breed was not possible given eight dogs. The resulting groups post simple randomization were unbalanced in breed; as such, one Labrador and one non‐Labrador were randomly selected to swap groups. After this pseudorandom procedure, the groups were relatively balanced for age and breed. For each day, each of the four dogs was randomized to a different treatment, i.e., each received naloxone, nalmefene, naltrexone, or saline once. Block randomization ensured that no two dogs received the same treatment during the same day.

The present study defined re‐narcotization as a sedation score greater than or equal to 10/14 at a point after a treatment (naloxone, naltrexone, or nalmefene) had already returned a score to baseline. Administration of naloxone hydrochloride (0.1 mg kg^−1^ IM) was performed if sedation scores were greater than or equal to 10/14 at 20 min following reversal administration (see Figure 1).

### 2.3 Sedation Scoring

Prior to placement of the intravenous catheter, a baseline sedation score was obtained utilizing a previously described subjective sedation scoring system with slight modification (Hofmeister et al. 2010). All observations were made while the dogs were loosely held on a leash by a known handler. A sedation score was obtained 5 min following methadone administration. Additional sedation scores were then obtained at the following intervals: immediately prior to reversal, 1 min post‐reversal, 5 min post‐reversal, 15 min post‐reversal, 30 min post‐reversal, 45 min post‐reversal, 60 min post‐reversal, and then at 60 min intervals until 6 h since reversal administration and prior to blood collection whenever possible. All observations were made by a single observer (TM) who was blinded to the reversal agent being administered (0.1 mg kg^−1^ naloxone hydrochloride, naltrexone hydrochloride, nalmefene base, or 0.1 mL kg^−1^ saline). At each time point, a video was obtained and reviewed by a second, blinded observer that was not present during the execution of the study (CB). Video clips were coded by the dog's name and a number not associated with the phase or sedation time period and were uploaded for review by the second observer.

### 2.4 Sample Collection

Blood was collected by an individual not involved in sedation scoring following methadone administration but prior to naloxone, naltrexone, nalmefene, or saline administration, and then at 1, 2, 5, 15, 30, 45, 60, and 120, 240, 360 min, as well as 24, 30, 48, 54, and 72 h after reversal or placebo administration. Samples were obtained from the catheter by withdrawing 1–2 mL of blood, discarding it, drawing 3 mL of blood, and placing it in a 5 mL vacutainer with lithium heparin (56 USP units). If blood was unable to be sampled from the preplaced catheter, it was obtained by direct puncture of the cephalic or saphenous veins. Samples were centrifuged for 10 min at 1000 *g*. Plasma was transferred to a cryovial and stored at 4°C until all samples were collected, then frozen at −80°C until analysis.

### 2.5 Measurement of Drug Concentrations

#### 2.5.1 Chemicals and Reagents

The reference standards of methadone (CAS #76‐99‐3), naloxone (CAS #465‐65‐6), naltrexone (CAS #16590‐41‐3), and isotopically labeled internal standards, *d*_*9*_‐methadone (CAS #1435933‐74‐6), *d*_*5*_‐naloxone (CAS #1261079‐38‐2), *d*_*3*_‐naltrexone (CAS # 1261080‐26‐5), were obtained from Cerilliant Corp. (Round Rock, TX, USA). The reference standards of nalmefene (CAS #55096‐26‐9) and isotopically labeled *d*_*3*_‐nalmefene (CAS # NA) were purchased from Toronto Research Chemicals (Toronto, ON, Canada). Methanol (MeOH, LC–MS grade), Methyl *tert*‐butyl ether (MTBE, HPLC grade), water (LC–MS grade), ammonium hydroxide (HPLC grade), formic acid (FA, ACS grade, 98%) and acetonitrile (LC–MS grade) were from EMD Millipore (Bedford, MA, USA).

#### 2.5.2 Sample Preparation

Liquid–liquid extraction (LLE) using MTBE was employed to extract all four analytes from the canine plasma samples. First, 10 μL of internal standard mixture was added to 0.05 mL of sample, and the sample was vortexed; then, 2 mL of ammonium hydroxide (pH 9.5) and 5 mL of MTBE were added to the sample. The sample tubes were mixed on a rotorack for 10 min. After that, the sample tubes were centrifuged at 1409 × *g* for 10 min. The top organic layer was transferred to a culture tube and was dried down at 60°C (Techni Dri‐Block DB‐3, Duxford, Cambridge, UK) under a steady stream of air. The dried extract was reconstituted in 500 μL of 5 mM NH_4_FA/FA:acetonitrile (90:10, v/v) and transferred into an appropriate insert. For LC–MS/MS analysis, 10 μL of the reconstituted extract was used.

#### 2.5.3 Liquid Chromatographic and Mass Spectrometric Conditions

Sample analysis was performed by an LC–MS/MS system, consisting of an ExionLC liquid‐chromatographic system and a Triple Quad 7500 triple stage quadrupole mass spectrometer equipped with an OptiFlow Pro electrospray ionization source (AB Sciex LLC, Framingham, MA, USA). LC separation was performed on a reversed‐phase ACE C18 column (75 × 2.1 mm, 5 μm) with an ACE guard column (10 × 2.1 mm, 5 μm; Mac‐Mod Analytical, Chadds Ford, PA, USA). Mobile phase A was 5 mM NH_4_FA/FA and mobile phase B was acetonitrile. The following mobile phase gradient was used for LC separation: 0 min, 90/10 (A/B); 0.1 min, 90/10 (A/B); 3.0 min, 30/70 (A/B); 3.1 min, 10/90 (A/B); 4.0 min, 10/90 (A/B); 4.1 min, 90/10 (A/B); 5.0 min, 90/10 (A/B). The mobile phase flow rate was 500 μL/min.

The mass spectrometer was operated in positive electrospray ionization mode. The source parameters were: ion source temperature 500°C, ion spray voltage 3000 V. Curtain gas, gas 1, and gas 2 were 40, 70, and 70 psi, respectively. The analytes were monitored by *multiple*‐*reaction monitoring* (*MRM*) mode. The MRM transitions monitored in this method for methadone, naloxone, naltrexone, and nalmefene were *m/z* 310→*m/z* 204, *m/z* 328→*m/z* 310, *m/z* 342→*m/z* 212, and *m/z* 240→*m/z* 268, respectively. For *IS* standards of *d*_*9*_‐methadone, *d*_*5*_‐naloxone, *d*_*3*_‐naltrexone, *d*_*3*_‐nalmefene, the MRM transitions monitored were *m/z* 319→*m/z* 162, *m/z* 333→*m/z* 315, *m/z* 345→*m/z* 270, and *m/z* 343→*m/z* 268, respectively. Data acquisition and processing were performed using SCIEX OS (v. 2.1.6, AB Sciex LLC, Framingham, MA, USA).

#### 2.5.4 Quantification of Analytes in Canine Plasma

Quantification of the analytes was conducted at the Pennsylvania Equine Toxicology and Research Laboratory, which is accredited by the American Association for Laboratory Accreditation and complied ISO 17025 International Guidelines (International Standards, ISO/IEC 17025, 2017, Geneva, Switzerland). The method was validated for specificity, sensitivity, linearity, matrix effect, recovery, accuracy and precision according to the United States Food and Drug Administration guidelines for validation of bioanalytical methods (“U.S. Department of Health and Human Services Food and Drug Administration, Bioanalytical Method Validation Guidance for Industry,” 2018). Validation data are provided as Tables S1A–D. Corresponding isotopically‐labeled internal standards (IS) were employed for quantification of analytes. The lower limit of quantification (LLOQ) validated were 0.1 ng mL^−1^, 0.02 ng mL^−1^, 0.02 ng mL^−1^ and 0.05/mL for methadone, naloxone, naltrexone, and nalmefene, respectively. Fresh calibration curves were prepared daily for sample analysis. The calibration curves were generated by plotting the ratios of peak areas of analytes to that of corresponding isotopically‐labeled ISs against analyte concentration. Linear regression with 1/*x* ^2^ weighting factor was used to describe the regression relationships. The linear quantification concentration ranges were 0.1–200, 0.02–20, 0.02–20 and 0.05–20 ng mL^−1^ for methadone, naloxone, naltrexone, and nalmefene, respectively. The coefficient of determination (*r* ^2^) was ≥ 0.99 for all calibration curves when quantifying the plasma samples. The samples with concentrations higher than the highest calibrator were diluted and re‐analyzed within the linear dynamic range of quantification.

### 2.6 Statistical Analysis

All analyses were conducted using Stata 17MP (StataCorp, TX, USA) with two‐sided tests of hypotheses and a *p*‐value < 0.05 as the criterion for statistical significance. Descriptive analyses include marginal means and standard errors, as well as median and range for age, as it was not normally distributed. Tests of normal distribution (Shapiro–Wilk test) were performed to determine the extent of skewness of the data. Frequency counts and percentages were used for categorical variables (e.g., sex, signalment and others). Inferential statistical analysis was conducted using robust linear regression. For each of the outcomes of interest, fixed effects were set as the statistical interaction between categorical time when the sample was obtained and treatment. Random effects were set on the level of individual animals. Marginal (model‐adjusted) means and effects were reported with their respective 95% confidence intervals unless otherwise specified. Fisher's protected least significant difference was used to control for multiple comparisons. *Post hoc* power analysis was performed with the following assumptions. It was assumed that the effect of the reversal agents would last the whole length of the experiment (360 min), with expected significant differences at 5, 30, and 60 min between the reversal agents. Assuming an alpha = 0.05 and a sample size of eight animals per group, it was estimated that a minimum difference of 5 min with a power of 1.0 could be detected. We were not able to detect a difference of 1 min, power = 0.46.

## 3 Results

The mean weight of the eight dogs was 28.2 ± 5.4 kg and did not significantly change across the four phases (*p* = 0.659). Due to behavioral reasons, one dog was excluded from the study after two phases. The remaining seven dogs completed all four phases, each receiving naloxone, naltrexone, nalmefene, or saline one time each. A total of 30 trials were completed. All eight dogs tolerated the methadone administration without vomiting or regurgitation. In the 30 trials, oxygen supplementation was provided in five instances. Oxygen supplementation was administered to three dogs once, and one dog twice. Bradycardia was observed in five of eight dogs (HR less than 60 beats per minute) prior to reversal; however, no dogs experienced hypotension (MAP < 60 mmHg). No bradycardia occurred following the administration of a reversal agent. Following methadone administration, six of eight dogs panted. No oxygen supplementation was necessary 5 min following intramuscular administration of naloxone, naltrexone, or nalmefene. No adverse events were observed with any of the reversal agents, and no dog that received a reversal agent required rescue naloxone administration. All dogs that received saline were administered naloxone at 20 min because sedation scores were greater than or equal to 10/14 at 20 min following reversal administration.

**FIGURE 2:**
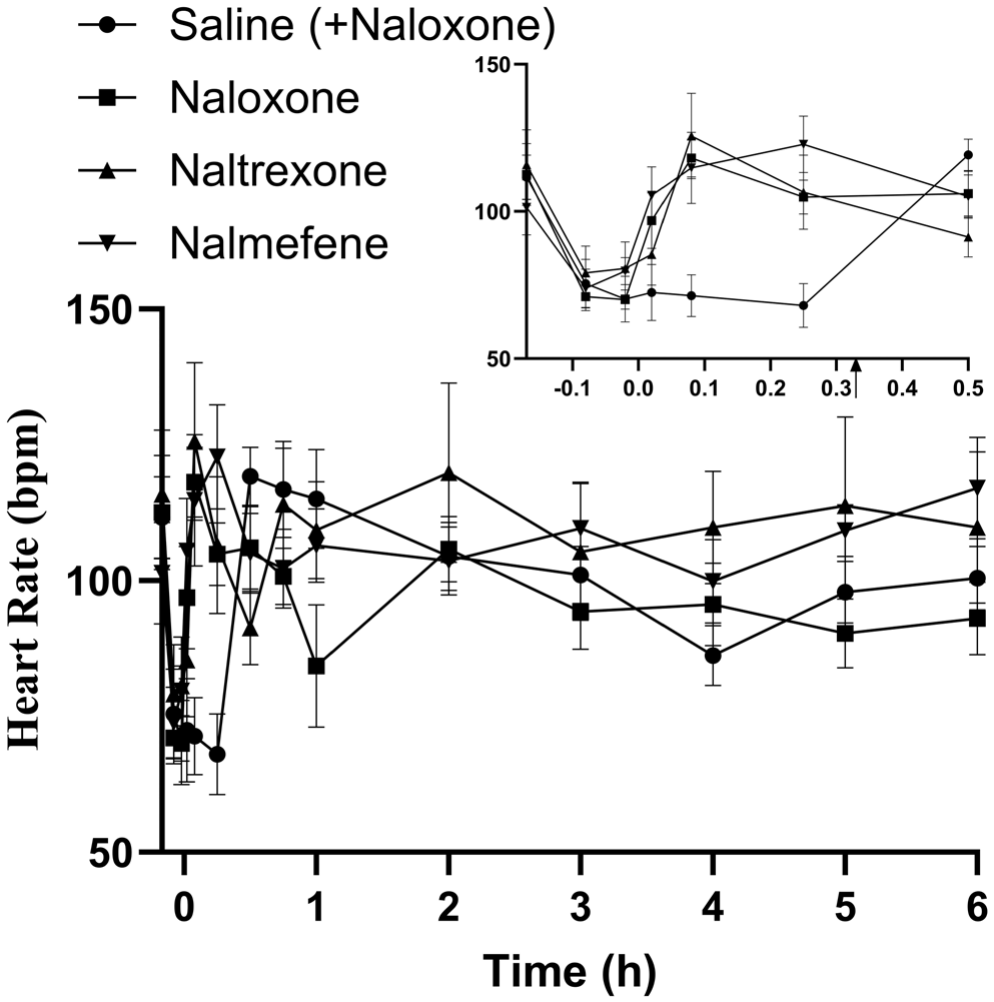
Marginal mean heart rate versus time data following the administration of methadone HCl (1 mg kg^−1^) intravenously to eight dogs on four different occasions with subsequent intramuscular administration of naloxone (*n* = 7), naltrexone (*n* = 8), and nalmefene (*n* = 8) at 0.1 mg kg^−1^, or saline with naloxone (0.1 mg kg^−1^) at 20 min (arrow in inset; *n* = 7). Data are presented as marginal mean ± SD for each treatment (different symbol per reversal). Inset enables visualization of the data between 0 and 0.5 h.

The marginal mean heart rates (beats per minute) over each time point are summarized in Figure 2 and are displayed in Table S2. The marginal mean HR significantly decreased from a baseline value of 110.3 ± 9.8–74.9 ± 7.2 bpm 5 min following methadone administration, a 32.1% decrease. The marginal mean HR between the three treatment groups was all significantly increased compared to saline by 5 min following reversal (*p* < 0.001) The marginal mean HR was increased in the nalmefene group (122.76 ± 9.65), 95% CI [103.9–141.7] compared to the naltrexone group (106.5 ± 12.7), 95% CI [81.7–131.3] at the 15‐min post‐reversal period (*p* = 0.014) and in the nalmefene group (117.05 ± 9.3), 95% CI [98.8, 135.3] compared to the naloxone group (93 ± 6.7), 95% CI [79.9, 106.1] at 6 h post‐reversal (*p* = 0.036). There were no significant differences between the three reversal agents at any other time point or between the saline and reversal groups following rescue naloxone administration.

**FIGURE 3:**
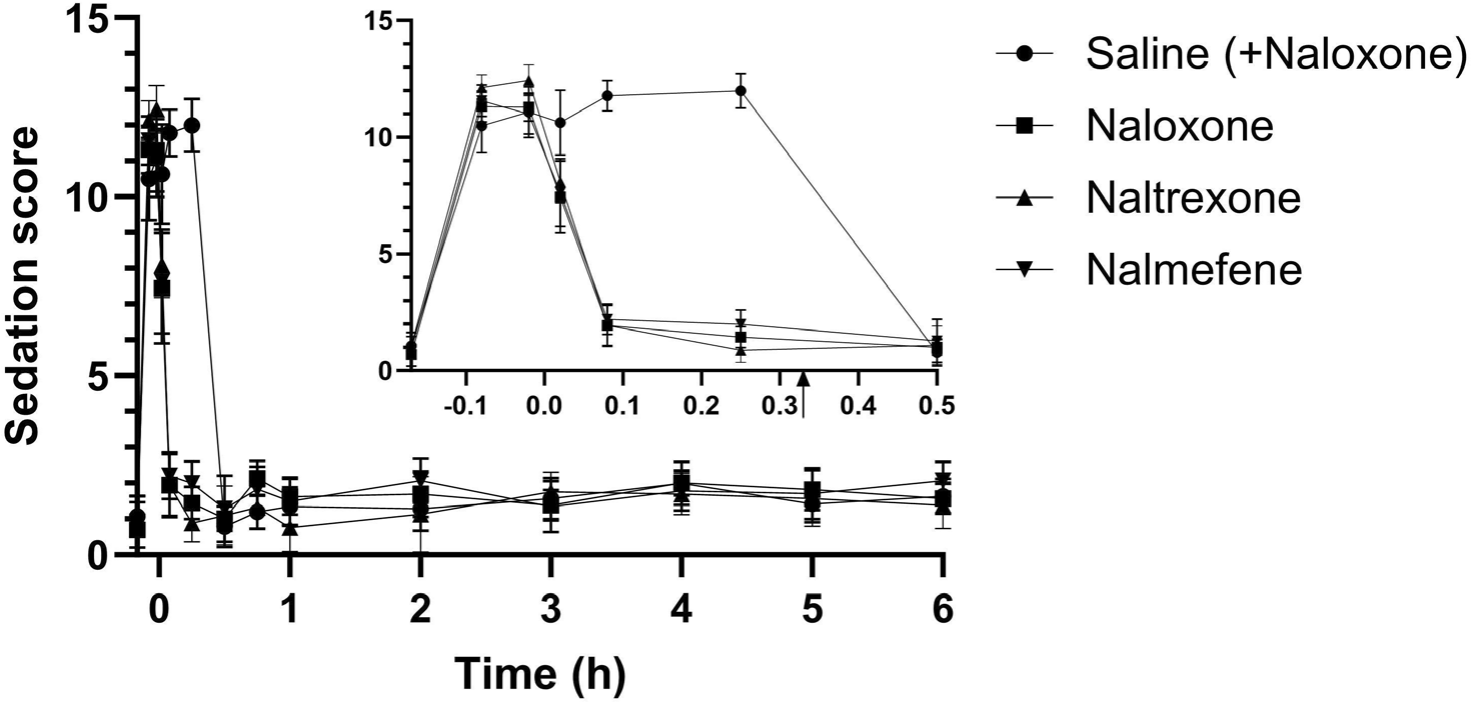
Marginal mean sedation score versus time data following the administration of methadone HCl (1 mg kg^−1^) intravenously to eight dogs on four different occasions with subsequent intramuscular administration of naloxone (*n* = 7), naltrexone (*n* = 8), and nalmefene (*n* = 8) at 0.1 mg kg^−1^, or saline with naloxone (0.1 mg kg^−1^) at 20 min (arrow in inset; *n* = 7). Data are presented as mean ± SD for each treatment (different symbol per treatment). Inset enables visualization of the data between 0 and 0.5 h.

The marginal mean sedation scores over time are summarized in Figure 3 and are displayed in Table S3. Across all groups, the mean sedation score prior to methadone was 0.93/14 and prior to reversal was 11.45/14. Mean sedation scores at 1 min post reversal were 7.6, 7.4, 8.1, and 10.6; and at 5 min were 2.2, 1.9, 1.9, and 11.8 for nalmefene, naloxone, naltrexone, and saline, respectively. There were no significant differences in sedation scoring between the two observers (*p* = 0.444). There were no statistically significant differences between treatment groups at baseline. At the time points 5 and 15 min post‐reversal, the marginal mean sedation scores of all three opioid reversal treatments were significantly decreased compared to saline (*p* < 0.001). After rescue naloxone was administered to the saline treatment group, there were no significant differences in sedation scores between the treatments at any of the remaining time points.

**FIGURE 4:**
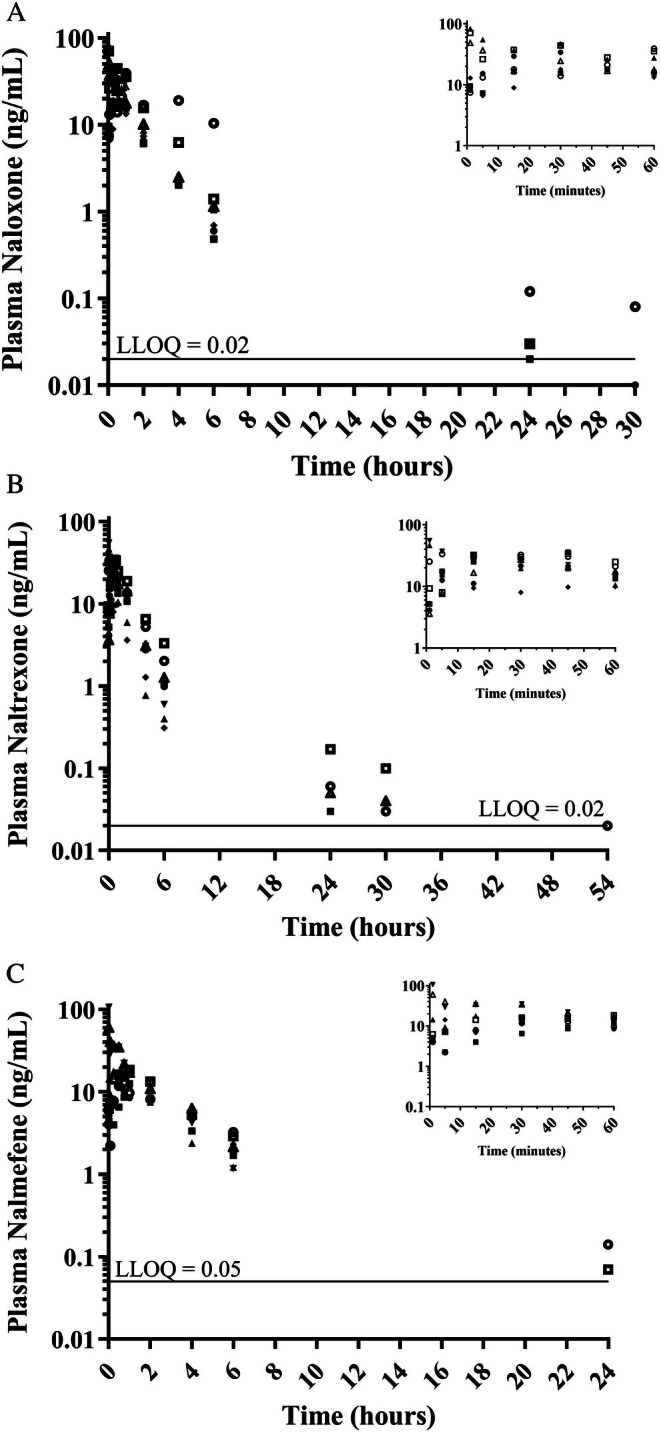
Plasma concentration versus time data following the intramuscular administration of naloxone HCl (A, *n* = 7), naltrexone HCl (B, *n* = 8), and nalmefene base (C, *n* = 8) at 0.1 mg kg^−1^ (different symbol per dog). The study was conducted as a 4‐phase randomized crossover with blocking in which each dog received the reversal agent or saline 5 min after methadone‐induced sedation (1 mg kg^−1^ IV). The horizontal line represents the lower limit of quantification (LLOQ) for the analytical method for each drug.

The plasma concentration of methadone and each reversal agent was measured for each trial during the first 6 h and between 24 and 72 h after their administration. Methadone was quantifiable in 15 out of 30 trials for all time points including 72 h. Substantially greater initial plasma concentrations were measured in six out of 30 trials (> 1000 ng mL^−1^; None in phase 1, Dogs 1, 2 and 6 in phase 2, Dogs 2 and 7 in phase 3, and Dog 6 in phase 4) and this did not correlate with the location of intravenous catheter placement (Appendix S1). No differences in sedation scores were observed for these trials. The plasma concentration versus time data were excluded from further analysis but are available for viewing in the Appendix S1 and should be interpreted cautiously. The reversal agents were quantifiable in all blood samples collected up to 6 h in all 30 trials. They were inconsistently detected at the next time point of 24 h and at all later time points (Figure 4, Table S4).

## 4 Discussion

Working dogs are frequently exposed to narcotics. This is the first study to evaluate the ability of three different reversal agents to reverse the effects of methadone, a long‐lasting opioid. Although methadone is not currently considered to be a hazardous compound in illicit street drugs, it was used to simulate exposure to a potentially lethal, long‐acting opioid (e.g., carfentanil). The dose of methadone used in this study resulted in clinically significant sedation, according to the sedation scoring system used in all trials conducted (Hofmeister et al. 2010). By the 5 min post‐reversal time point, dogs that received an opioid reversal agent (naloxone, naltrexone, or nalmefene) had significantly decreased sedation scores and subsequent sedation scores were not significantly different compared to baseline for the 6 h study period. No adverse events were observed with any of the reversal agents. These findings indicate that all three reversal agents administered at 0.1 mg kg^−1^ intramuscularly rapidly, reliably, safely, and unequivocally antagonized the methadone‐induced sedation in this group of working dogs. Furthermore, all dogs that received saline met the criteria for receiving naloxone (IM) at the 20‐min time point to reverse the ongoing methadone‐induced sedation. There were no statistically significant differences between sedation scores of treatment groups from 30 min to the conclusion of the scoring at 6 h, indicating that re‐narcotization was not observed in this study.

Previous studies have evaluated the efficacy of the μ‐antagonists naloxone and naltrexone; however, this is the first canine study to look at the qualitative effects of the reversal of opioid‐induced sedation using IM nalmefene. Dyson et al. found that intravenously administered nalmefene (0.03 mg kg^−1^) or naloxone (0.4 or 1.2 mg; 0.009–0.038 mg kg^−1^) reversed oxymorphone‐induced sedation in dogs (0.1–0.14 mg kg^−1^ IV; Dyson et al. 1990). Interestingly, 2 h post‐reversal, mild re‐narcotization was seen in the naloxone‐treated dogs, but not in the nalmefene‐treated dogs. Intravenous oxymorphone in dogs can cause sedation lasting up to 3 h (Dhillon and Paul 1973), and the absence of re‐narcotization suggested that nalmefene may be superior to naloxone as a reversal agent for longer acting narcotics when administered intravenously at lower doses than those used in the current study.

The dose of methadone chosen for this study (1 mg kg^−1^ IV) has been shown to cause sedation for 2–3 h (Maiante et al. 2009; Menegheti et al. 2014). However, in the present study, re‐narcotization was not observed following any of the reversal treatments, with no significant increase in sedation score or recumbency noted following administration of the reversal agent. Dyson et al. detected mild re‐narcotization following administration of naloxone to dogs following oxymorphone sedation; however, the dose of naloxone used in the present study was higher than what Dyson et al. used (0.009–0.038 mg kg^−1^ vs. 0.1 mg kg^−1^; Dyson et al. 1990). The effect of this higher dose in preventing re‐narcotization is uncertain.

In another study, Wilhelm et al. described the pharmacodynamics of nalmefene and naloxone in dogs when administered as an IV infusion (12 and 48 μg/kg/h for 30 min, respectively) to reverse respiratory depression induced by a concomitant fentanyl IV infusion (30 μg/kg/h for 7 h). They assessed their ability to reverse the opioid‐induced respiratory depression at equipotent doses. Nalmefene had a longer duration of action than naloxone, suggesting it may be a better choice to reverse the effects of long‐acting opioids (Wilhelm et al. 1995). Freise et al. (2012) also found that dogs re‐narcotized within 1–3 h following intramuscular naloxone administration (0.04–0.16 mg kg^−1^) while receiving a long‐acting fentanyl patch overdose of 13 mg kg^−1^. The present study, however, was unable to demonstrate advantages of one reversal agent over another in working dogs due to the lack of re‐narcotization.

The heart rates of the dogs in this study decreased by 32.1% 5 min following methadone administration, which is comparable with previous studies by Menegheti et al. (2014) and Maiante (2009) in which heart rates decreased by 15%–33% and 32%–46% respectively (Maiante et al. 2009; Menegheti et al. 2014). In the present study following opioid reversal, the marginal mean heart rate was significantly increased at 5 and 15 min post‐reversal with either naloxone, naltrexone, or nalmefene compared to saline (*p* < 0.001) and was not significantly different from baseline. This suggests that all may be effective in the urgent care treatment of decreases in heart rate due to opioid exposure in a working dog field exposure scenario. The statistically significant difference of the marginal heart rate in beats per minute between nalmefene (117.05 ± 9.3; 95% CI [98.8, 135.3]) and naloxone (93 ± 6.7; 95% CI [79.9, 106.1]) at 6 h post‐reversal (*p* = 0.036) may suggest that nalmefene antagonizes the effect of decreases in heart rate caused by opioids longer than naloxone, though additional research including electrocardiogram analysis is warranted.

There are multiple previous pharmacokinetic studies of methadone in dogs. Following 1 or 0.5 mg kg^−1^ IV administration of methadone to dogs, Kukanich et al. (2005) found the half‐life of methadone to be 1.75 ± 0.25 h in beagles and 1.53 ± 0.18 h in greyhounds (KuKanich and Borum 2008), respectively. In the present study, while the elimination profiles appear similar to previous studies, the integrity of the methadone data was questioned due to the presence of spuriously high values in 6 of the 30 trials. The cause of these spuriously high values has not been determined. Variability from one trial to another has been reported previously in a dog (Garrett et al. 1985). Following administration of a 2.24 mg kg^−1^ intravenous injection of methadone in the same dog at bimonthly intervals for four experiments, Garrett et al. reported calculated macro‐rate constant A values of 459, 3491, 1408, and 760 ng mL^−1^. The lack of a consistent pattern in their experiments and in the present study does not provide a clear explanation for how or why this variability in methadone concentration was observed during the early collection times. In our study, the samples were analyzed multiple times and the data thoroughly interrogated to ensure the values were accurately determined for the sample; however, sampling and processing errors cannot be excluded. Additionally, the use of the same vein, despite using a different catheter, could have contributed to higher values in some trials; however, not all of them. In three of the six trials with spuriously high values, a different vein was used to collect the samples. These data have been included in the Appendix S1 for completeness and transparency; however, they should be interpreted with caution.

There are also previous pharmacokinetic studies for naloxone, naltrexone, and nalmefene in dogs at various doses and routes of administration. The half‐lives reported for naloxone following IV dosing include 1.19 ± 0.15 (5 mg kg^−1^ IV; Pace et al. 1979) and 0.62 ± 0.11 h (0.04 mg kg^−1^ IV; Pace et al. 1979; Wahler et al. 2019). Following IM naloxone dosing, Papich and Narayan (2022) reported a half‐life of 1.20 ± 0.86 h for a dose of 0.04 mg kg^−1^. Pace et al. reported a half‐life of 1.42 ± 0.15 h for naltrexone administered by the IV route (5 mg kg^−1^). For nalmefene, Papich and Narayan (2022) reported a half‐life of 1.39 ± 1.18 h for a dose of 0.014 mg kg^−1^ IM. Gaekens et al. (2016) reported mean half‐lives for three dogs of 0.85 h for a dose of 0.3 mg kg^−1^ IV nalmefene and 0.92 h for a dose of 0.02 mg kg^−1^ IM nalmefene; however, limited data points were available following IM administration in their study.

In the present study, working dog availability restricted sample collection to the first 6 h and then at 24, 30, 48, 54, and 72 h after the drug administrations. Interestingly, samples collected between 24 and 72 h demonstrated that in some dogs there was evidence of a slower terminal elimination phase for the reversal agents. The increased sensitivity afforded by the analytical method and instrumentation employed allowed for the detection of a “deep compartment” (i.e., a compartment with a slower elimination rate) that has not been previously reported. While the data in this study were too sparse to provide reliable pharmacokinetic estimates for this new terminal phase, future studies with samples collected between 6 and 24 h using comparable methodology would enable estimation of this slower elimination rate constant.

There were several additional limitations of the study. The sedation scoring system, while validated, was subjective and may have lacked sensitivity, reducing the potential for identification of subtle differences between groups, especially in the time period immediately following opioid reversal. To address the subjective nature of the scoring, in addition to live scoring by a trained and blinded individual, videos blinded to group and time were reviewed by a second observer. Despite the limitations of video scoring, the scores between the two observers were not significantly different. The scores could have been confounded by the dogs' prior training to stand still and ignore distractions, (i.e., dogs appearing more sedate), or by the nature of working dogs' high energy (i.e., dogs appearing less sedate); however, the cross‐over design reduced the effect of an individual dog's behavior. Parallel to obtaining sedation scores, blood collection sometimes occurred. Sedation scores may have been lower in instances where simultaneous sedation scoring and blood collection occurred; however, blood collection was typically obtained from a catheter and dogs had been trained to stand still for the procedure.

There is also the limitation of using the same relatively high dosage on a mg kg^−1^ basis of the three reversal agents despite different potencies. While one would predict this to result in lower sedation scores for nalmefene, as it is reportedly four times more potent than naloxone, there was no significant difference in sedation scores between treatments, and all treatments significantly reduced sedation scores as compared to saline. For the present study, a dose of 1 mg kg^−1^ methadone IV was chosen because of its longer duration of action than fentanyl, and this dose was thought to provide the potential for re‐narcotization; however, re‐narcotization was not observed, presumably due to the relatively high doses administered of the three reversal agents. Also, a limitation of the study may have been the use of methadone. Methadone was selected for its longer duration of action compared with fentanyl, but it may not accurately reflect the cardiorespiratory effects of a more potent opioid (such as fentanyl or carfentanil) to which a working dog is more likely to be exposed. Fentanyl and carfentanil are 50–100 times and 10,000 times more potent than morphine, respectively, with fentanyl having a shorter duration of action and a higher binding affinity for the mu opioid receptor than methadone (Ellis 2018; Drug Enforcement Agency 2020; Zawilska et al. 2021). The analgesic potency of methadone is similar to morphine, though methadone is more lipophilic, is eliminated more slowly, and causes greater cardiovascular depression at equivalent dosages (Kukanich and Papich 2009). When administered at 1 mg kg^−1^ IV in dogs, it was observed that methadone caused a greater and more prolonged reduction in heart rate than morphine, but that methadone sedation waned around 2–3 h after administration without reversal (Maiante et al. 2009). Therefore, the window for observation of re‐narcotization opportunity after reversal may have been narrow with methadone at this dosage, and a longer acting opioid or an opioid infusion may be necessary to detect differences in clinical effect between the three antagonists, if existent.

Bailey et al. administered doses of 3 mg kg^−1^ fentanyl IV to dogs, which did not result in respiratory or cardiac arrest; the LD50 of fentanyl in dogs is reported as 14 and 29 mg kg^−1^ for methadone (Bailey et al. 1987; Kase et al. 1959; FDA Fentanyl Citrate 2013; Kukanich and Papich 2009). Despite the relatively high doses of clinically used opioids required to cause cardiovascular or respiratory arrest as compared with humans, exposure of working dogs to opioids remains a risk that may impact their welfare, result in sedation, or otherwise affect their ability to perform their work safely and effectively.

The tested opioid reversal agents were similar to one another with respect to methadone in the present study; however, these results should be interpreted with caution. Their efficacy following exposure to higher potency opioids cannot be assured by this study given the differences in duration of action, binding affinity, and potency between methadone and the more potent opioids a working dog may encounter. This study evaluated a single dose of methadone in dogs that induced deep sedation. Further testing with longer lasting and ultra‐potent opioids, as well as additional doses, would be needed to determine if the results of the present study are broadly applicable.

## 5 Conclusion

This study showed that intramuscular administration of naloxone, naltrexone, or nalmefene all rapidly reverse 1 mg kg^−1^ IV methadone sedation in working dogs with no evidence of adverse effects or re‐narcotization occurring within 6 h. Naloxone is currently used as a first‐line agent for opioid reversal following working dog exposure. While all reversal agents were well‐tolerated and effective in reversal, the present study did not detect a clinical reason to replace naloxone with naltrexone or nalmefene. Law enforcement officers and first responders that already carry naloxone can utilize naloxone as a reversal agent for working dogs exposed to opioids in the field but should follow up with appropriate veterinary care after proper decontamination of their dog. Further studies are necessary to determine the optimal reversal agent for ultra‐potent or long‐acting opioids.

## Author Contributions

M.A.R., C.M.O., and A.M. designed the study and obtained funding for the study. T.M., K.S., and C.M.O. were blinded and collected the samples and data. T.M. and C.B. performed the blinded sedation scoring. Y.Y. and R.P. developed the analytical methods and analyzed the samples in a blinded manner to determine drug concentrations. T.M. and M.A.R. performed data analysis, composed the figures and wrote the first draft of the manuscript. D.S. performed the statistical analyses. All authors revised and approved the final manuscript.

## Ethics Statement

The animal welfare standards set forth by the United States were met for the inclusion of these working dogs in this study, and the ethical policies of this journal were adhered to in accordance with the journal guidelines. This protocol was approved by the Institutional Animal Care and Use Committee at the University of Pennsylvania (Protocol # 807164) and the Army Animal Care and Use Review Office Proposal (Number 77711‐ST).

## Conflicts of Interest

The authors declare no conflicts of interest.

## Supporting information


Data S1.

Appendix S1.

## Data Availability

Data included in this manuscript are available upon request.
