# Supplementary material for: A Randomized, Blinded, Placebo‐Controlled Crossover Study of the Pharmacokinetics and Pharmacodynamics of Naloxone, Naltrexone, and Nalmefene in Methadone‐Sedated Working Dogs
Source: J Vet Pharmacol Ther. 2025 May 8;48(5):359–67. doi: 10.1111/jvp.13515 (PMC12415812; doi:10.1111/jvp.13515)
Supplement: Supplementary file 1 — Data S1. [file JVP-48-359-s001.docx]

| Supplemental Table 1A. Validation Summary of Methadone Quantification in Dog Plasma^1^ | | | |
| --- | --- | --- | --- |
| Validation parameters | Fortified concentration (ng/mL) |  | Validation  results |
| Calibration curve  (n = 6) | 0.1 (LLOQ^2^)  0.2-200 | Percent Residual Accuracy^3^ (%) | 99.9  92.6-112.9 |
|  | 0.1-200 | Coefficient of determination (*R^2^*) | 0.9901 ± 0.0044 |
| Intra-assay  Accuracy and Precision  (4 conc., n = 6) | 0.1 (LLOQ) | Accuracy (%)  CV (%) | 100.7  9.9 |
|  | 5, 100, 150 | Accuracy (%)  CV (%) | 95.9-112.9  5.4-8.6 |
| Inter-assay  Accuracy and Precision  (4 conc., n = 10, 3 days) | 0.1 (LLOQ) | Accuracy (%)  CV (%) | 114.0  14.7 |
|  | 5, 100, 150 | Accuracy (%)  CV (%) | 96.0-102.9  4.6-11.7 |
| Matrix effect^4^  (3 conc., n = 6) | 5, 100, 150 | (%) | 83.1-102.1 |
| Extraction recovery^5^  (3 conc., n = 6) | 5, 100, 150 | (%) | 87.8-93.4 |
| ^1^ Method validation and sample analysis were performed according to the guidance of the US Food and Drug Administration (FDA) for Bioanalytical Method Validation (FDA, 2018). Accuracy was determined by comparing the mean of measured concentrations to the nominal concentrations, with an acceptable range of 85-115% (80-120% at the LLOQ level). Precision was measured by calculating the coefficient of variation (CV) of the measured concentrations, which needed to be within 15% (20% at the LLOQ level) (FDA, 2018).  ^2^ LLOQ: The lowest concentration of an analyte validated according to FDA guidance (FDA, 2018).  ^3^ Percent Residual Accuracy: The average accuracy of calibrators from multiple analyses across different concentrations (Logue and Manandhar, 2018).  ^4^ Matrix effect was assessed by comparing the peak areas of the analyte added into added to plasma extracts with the peak areas of the analyte spiked into the reconstitution solution  ^5^ Extraction recovery was determined by comparing the peak areas of the fortified samples to those of the analyte added to plasma extracts.  U.S. Department of Health and Human Services Food and Drug Administration, Bioanalytical Method Validation Guidance for Industry, 2018. <https://www.fda.gov/regulatory-information/search-fda-guidance-documents/bioanalytical-method-validation-guidance-industry> (Accessed: 10/17/2024)  B.A. Logue, E. Manandhar, Percent residual accuracy for quantifying goodness-of-fit of linear calibration curves, Talanta, 189 (2018) 527-533. | | | |

| Supplemental Table 1B. Validation Summary of Naloxone Quantification in Dog Plasma^1^ | | | |
| --- | --- | --- | --- |
| Validation parameters | Fortified concentration (ng/mL) |  | Validation  results |
| Calibration curve  (n = 6) | 0.02 (LLOQ^2^)  0.05-20 | Percent Residual Accuracy^3^ (%) | 94.2  93.6-111.8 |
|  | 0.02-20 | Coefficient of determination (*R^2^*) | 0.9901 ± 0.0044 |
| Intra-assay  Accuracy and Precision  (4 conc., n = 6) | 0.02 (LLOQ) | Accuracy (%)  CV (%) | 91.7  14.5 |
|  | 0.5, 10, 15 | Accuracy (%)  CV (%) | 100.3-111.9  4.0-6.4 |
| Inter-assay  Accuracy and Precision  (4 conc., n = 10, 3 days) | 0.02 (LLOQ) | Accuracy (%)  CV (%) | 97.5  5.4 |
|  | 0.5, 10, 15 | Accuracy (%)  CV (%) | 92.3-105.5  2.8-10.0 |
| Matrix effect^4^  (3 conc., n = 6) | 0.5, 10, 15 | (%) | 79.6-100.7 |
| Extraction recovery^5^  (3 conc., n = 6) | 0.5, 10, 15 | (%) | 84.9-92.3 |
| ^1^ Method validation and sample analysis were performed according to the guidance of the US Food and Drug Administration (FDA) for Bioanalytical Method Validation (FDA, 2018). Accuracy was determined by comparing the mean of measured concentrations to the nominal concentrations, with an acceptable range of 85-115% (80-120% at the LLOQ level). Precision was measured by calculating the coefficient of variation (CV) of the measured concentrations, which needed to be within 15% (20% at the LLOQ level) (FDA, 2018).  ^2^ LLOQ: The lowest concentration of an analyte validated according to FDA guidance (FDA, 2018).  ^3^ Percent Residual Accuracy: The average accuracy of calibrators from multiple analyses across different concentrations (Logue and Manandhar, 2018).  ^4^ Matrix effect was assessed by comparing the peak areas of the analyte added into added to plasma extracts with the peak areas of the analyte spiked into the reconstitution solution  ^5^ Extraction recovery was determined by comparing the peak areas of the fortified samples to those of the analyte added to plasma extracts.  U.S. Department of Health and Human Services Food and Drug Administration, Bioanalytical Method Validation Guidance for Industry, 2018. <https://www.fda.gov/regulatory-information/search-fda-guidance-documents/bioanalytical-method-validation-guidance-industry> (Accessed: 10/17/2024)  B.A. Logue, E. Manandhar, Percent residual accuracy for quantifying goodness-of-fit of linear calibration curves, Talanta, 189 (2018) 527-533. | | | |

| Supplemental Table 1C. Validation Summary of Naltrexone Quantification in Dog Plasma^1^ | | | |
| --- | --- | --- | --- |
| Validation parameters | Fortified concentration (ng/mL) |  | Validation  results |
| Calibration curve  (n = 6) | 0.02 (LLOQ^2^)  0.05-20 | Percent Residual Accuracy^3^ (%) | 93.0  93.7-113.3 |
|  | 0.02-20 | Coefficient of determination (*R^2^*) | 0.9850 ± 0.0104 |
| Intra-assay  Accuracy and Precision  (4 conc., n = 6) | 0.02 (LLOQ) | Accuracy (%)  CV (%) | 101.7  19.1 |
|  | 0.5, 10, 15 | Accuracy (%)  CV (%) | 99.3-108.3  2.9-8.8 |
| Inter-assay  Accuracy and Precision  (4 conc., n = 10, 3 days) | 0.02 (LLOQ) | Accuracy (%)  CV (%) | 98.3  19.7 |
|  | 0.5, 10, 15 | Accuracy (%)  CV (%) | 94.3-110.7  5.0-111.8 |
| Matrix effect^4^  (3 conc., n = 6) | 0.5, 10, 15 | (%) | 84.1-98.4 |
| Extraction recovery^5^  (3 conc., n = 6) | 0.5, 10, 15 | (%) | 90.5-95.1 |
| ^1^ Method validation and sample analysis were performed according to the guidance of the US Food and Drug Administration (FDA) for Bioanalytical Method Validation (FDA, 2018). Accuracy was determined by comparing the mean of measured concentrations to the nominal concentrations, with an acceptable range of 85-115% (80-120% at the LLOQ level). Precision was measured by calculating the coefficient of variation (CV) of the measured concentrations, which needed to be within 15% (20% at the LLOQ level) (FDA, 2018).  ^2^ LLOQ: The lowest concentration of an analyte validated according to FDA guidance (FDA, 2018).  ^3^ Percent Residual Accuracy: The average accuracy of calibrators from multiple analyses across different concentrations (Logue and Manandhar, 2018).  ^4^ Matrix effect was assessed by comparing the peak areas of the analyte added into added to plasma extracts with the peak areas of the analyte spiked into the reconstitution solution  ^5^ Extraction recovery was determined by comparing the peak areas of the fortified samples to those of the analyte added to plasma extracts.  U.S. Department of Health and Human Services Food and Drug Administration, Bioanalytical Method Validation Guidance for Industry, 2018. <https://www.fda.gov/regulatory-information/search-fda-guidance-documents/bioanalytical-method-validation-guidance-industry> (Accessed: 10/17/2024)  B.A. Logue, E. Manandhar, Percent residual accuracy for quantifying goodness-of-fit of linear calibration curves, Talanta, 189 (2018) 527-533. | | | |

| Supplemental Table 1D. Validation Summary of Nalmefene Quantification in Dog Plasma^1^ | | | |
| --- | --- | --- | --- |
| Validation parameters | Fortified concentration (ng/mL) |  | Validation  results |
| Calibration curve  (n = 6) | 0.05 (LLOQ^2^)  0.1-20 | Percent Residual Accuracy^3^ (%) | 96.6  88.2-105.0 |
|  | 0.05-20 | Coefficient of determination (*R^2^*) | 0.9860 ± 0.0036 |
| Intra-assay  Accuracy and Precision (4 conc., n = 6) | 0.05 (LLOQ) | Accuracy (%)  CV (%) | 99.3  12.1 |
|  | 0.5, 10, 15 | Accuracy (%)  CV (%) | 96.9-112.6  6.0-7.4 |
| Inter-assay Accuracy and Precision  (4 conc., n = 10, 3 days) | 0.05 (LLOQ) | Accuracy (%)  CV (%) | 91.7  10.4 |
|  | 0.5, 10, 15 | Accuracy (%)  CV (%) | 87.5-100.3  3.0-12.3 |
| Matrix effect^4^  (3 conc., n = 6) | 0.5, 10, 15 | (%) | 77.1-95.0 |
| Extraction recovery^5^  (3 conc., n = 6) | 0.5, 10, 15 | (%) | 90.2-93.0 |
| ^1^ Method validation and sample analysis were performed according to the guidance of the US Food and Drug Administration (FDA) for Bioanalytical Method Validation (FDA, 2018). Accuracy was determined by comparing the mean of measured concentrations to the nominal concentrations, with an acceptable range of 85-115% (80-120% at the LLOQ level). Precision was measured by calculating the coefficient of variation (CV) of the measured concentrations, which needed to be within 15% (20% at the LLOQ level) (FDA, 2018).  ^2^ LLOQ: The lowest concentration of an analyte validated according to FDA guidance (FDA, 2018).  ^3^ Percent Residual Accuracy: The average accuracy of calibrators from multiple analyses across different concentrations (Logue and Manandhar, 2018).  ^4^ Matrix effect was assessed by compari^1^ ng the peak areas of the analyte added into added to plasma extracts with the peak areas of the analyte spiked into the reconstitution solution  ^5^ Extraction recovery was determined by comparing the peak areas of the fortified samples to those of the analyte added to plasma extracts.  U.S. Department of Health and Human Services Food and Drug Administration, Bioanalytical Method Validation Guidance for Industry, 2018. <https://www.fda.gov/regulatory-information/search-fda-guidance-documents/bioanalytical-method-validation-guidance-industry> (Accessed: 10/17/2024)  B.A. Logue, E. Manandhar, Percent residual accuracy for quantifying goodness-of-fit of linear calibration curves, Talanta, 189 (2018) 527-533. | | | |

**Supplemental Table 2.** Marginal mean heart rates (in beats per minute) for all dogs at each timepoint as well as the *p*-values comparing each treatment.

**Supplemental Table 3** Combined marginal mean sedation scores for both observers all dogs at each timepoint as well as the *p*-values comparing each treatment to another.

Supplemental Table 4. Naloxone, Naltrexone and Nalmefene Plasma Concentration vs Time Data

The LLOQ was 0.02 ng/mL for Naloxone and Naltrexone. The LLOQ was 0.05 ng/mL for Nalmefene. -- indicates that a sample was not collected.
