# Supplementary material for: A Randomized, Blinded, Placebo‐Controlled Crossover Study of the Pharmacokinetics and Pharmacodynamics of Naloxone, Naltrexone, and Nalmefene in Methadone‐Sedated Working Dogs
Source: J Vet Pharmacol Ther. 2025 May 8;48(5):359–67. doi: 10.1111/jvp.13515 (PMC12415812; doi:10.1111/jvp.13515)
Supplement: Supplementary file 2 — Appendix S1. [file JVP-48-359-s002.docx]

**Appendix**

**Sedation Scoring** (Hofmeister et al. 2010)*

| **Observation** | **Score** | **Description** |
| --- | --- | --- |
| Vocalization | 0 | Quiet |
|  | -1 | Whining softly but quiets with soothing touch |
|  | -2 | Whining continuously |
|  | -3 | Barking continuously |
|  |  |  |
| Posture | 3 | Lateral recumbency |
|  | 2 | Sternal recumbency |
|  | 1 | Sitting or ataxic while standing |
|  | 0 | Standing |
|  | -1 | Moving continuously |
|  |  |  |
| Appearance | 3 | Eyes sunken, glazed or unfocused; ventromedial rotation |
|  | 2 | Eyes glazed but follow movement |
|  | 1 | Protrusion of nictitating membrane; normal visual responses |
|  | 0 | Normal appearance |
|  | -1 | Pupils dilated; abnormal facial expression |
|  |  |  |
| Interactive behavior | 3 | Recumbent; no response to voice or touch |
|  | 2 | Recumbent; lifts head in response to voice or touch |
|  | 1 | Recumbent but stands in response to voice or touch |
|  | 0 | Standing or sitting up; normal response to voice or touch |
|  | -1 | Moves away from voice or touch; appears anxious |
|  | -2 | Growls or hisses when approached or touched |
|  | -3 | Bites or swats when approached |
|  |  |  |
| Restraint | 2 | Lies on floor with minimal restraint needed |
|  | 1 | Lies on floor with light restraint of head or neck |
|  | 0 | Sits up on floor, attempts to jump despite restraint |
|  | -1 | Struggles continuously against restraint |
|  | -2 | Cannot be restrained for > 20 seconds |
|  |  |  |
| Response to noise | 3 | No response to a hand clap near the head |
|  | 2 | Minimal response to a hand clap |
|  | 1 | Slow or moderate response to a hand clap near the head |
|  | 0 | Brisk response to a hand clap near the head; raises the head with eyes open |

*We would seek permission for the reproduction of this table if the paper is selected for publication.

**Appendix**

Methadone Plasma Concentration vs Time Data. Data should be interpreted with caution as sampling/processing errors cannot be ruled out. The LLOQ was 0.1 ng mL^-1^. <LLOQ = below the lower limit of quantification. -- indicates that a sample was not collected. * denotes trials in which methadone injection via butterfly catheter and subsequent IV catheter placement were in different veins.
